# Blocking-Free ELISA Using a Gold Nanoparticle Layer Coated Commercial Microwell Plate

**DOI:** 10.3390/s18103537

**Published:** 2018-10-19

**Authors:** Ruijia Huang, Ke Zhang, Guoshuai Zhu, Zhencheng Sun, Songliang He, Wenwen Chen

**Affiliations:** Guangdong Key Laboratory for Biomedical Measurements and Ultrasound Imaging, School of Biomedical Engineering, Shenzhen University Health Science Center, Shenzhen 518060, China; 2014222058@email.szu.edu.cn (R.H.); 2172243136@email.szu.edu.cn (K.Z.); 2172243164@email.szu.edu.cn (G.Z.); sunzhencheng2017@email.szu.edu.cn (Z.S.); 2161220129@email.szu.edu.cn (S.H.)

**Keywords:** gold nanoparticles, blocking-free, ELISA

## Abstract

Enzyme-linked immunosorbent assays (ELISA) show extensive application in immunoassays, to detect and monitor protein biomarkers in clinical diagnosis. Nevertheless, the time required and its multiple steps limit its application. We take advantage of a polyethyleneimine (PEI) gold nanoparticle (GNP) coated microwell plate to perform blocking-free ELISA, in which no nonspecific protein adsorption appears on the GNP layer. If the PEI-GNP coated microwell plate and immobilization of captured antibodies on the plate are prepared in advance, such as using an ELISA kit, the whole ELISA process can be finished in less than 2 h. Meanwhile, we have ensured that the GNP layer can preserve the precision and good linearity of ELISA without causing negative effects on the plate.

## 1. Introduction

Proteins are macromolecules performing a vast array of functions within organisms [1]. Some proteins are biomarkers related to different kinds of diseases and detection and monitoring of their concentrations are of great significance for clinical diagnosis [2,3,4]. Immunoassays are the most efficient strategy to detect proteins, in which the enzyme-linked immunosorbent assay (ELISA) is extensively applied [5,6,7]. ELISA combines antigens, specific antibodies, and enzymes covalently linked with antibodies. Due to the specific interactions between antigen and antibody, ELISA presents high precision and good linearity [8,9]. Nevertheless, poor blocking efficiency and a requirement for time-consuming, multiplex steps limit its application in clinics [10,11,12]. Taking a “sandwich” ELISA as an example, the process of primary antibody incubation, blocking antigens, enzyme labelled antibody incubation, and colorimetric substrate addition cannot be simplified. Blocking is a step for filling a plate to avoid unspecific adsorption of enzyme-labelled antibodies after primary antibodies adsorb on the plate [13,14]. As a consequence, research in the field of improving blocking efficiency or developing a blocking-free ELISA, without false positive results, have attracted a great deal of attention. Albumin from bovine serum (BSA), polyethylene glycol (PEG), and skimmed milk powder are the most widely used blocking agents [15,16,17]. Their type, concentration, and incubation time influence blocking efficiency a great deal. Even after extensive optimization, blocking will succeed, but 1 or 2 h are still essential.

It is known that the properties of a substrate are a critical factor in influencing protein adsorption in ELISA [18,19]. Therefore, substrate surface modifications could be the most valid means for exhibiting improvements over conventional ELISA. With recent advances in nanomaterials and polymer science, different strategies based on these are applied to an ELISA to increase sensitivity, blocking efficiency, or realize a blocking-free ELISA [20,21,22]. Commercial microwell plates with an in situ synthesized gold nanoparticle layer could amplify the signal and lower the detection limit of ELISA [23]. However, unspecific adsorption is detrimental in promoting sensitivity and is sometimes unavoidable. This method lacks exploration in improving blocking efficiency to decrease unspecific adsorption. Other researchers have made use of a layer-by-layer (LbL) self-assembly technique to fabricate polyelectrolyte multilayers on a polystyrene (PS) plate, which could inhibit nonspecific adsorption, even without blocking reagent adsorption [18]. Nonetheless, with the physical adsorption of antibodies on the functionalized surface of the plate, linearity of the calibration curve was poor and the greatest R^2^ value was only 0.92.

**Scheme 1:**
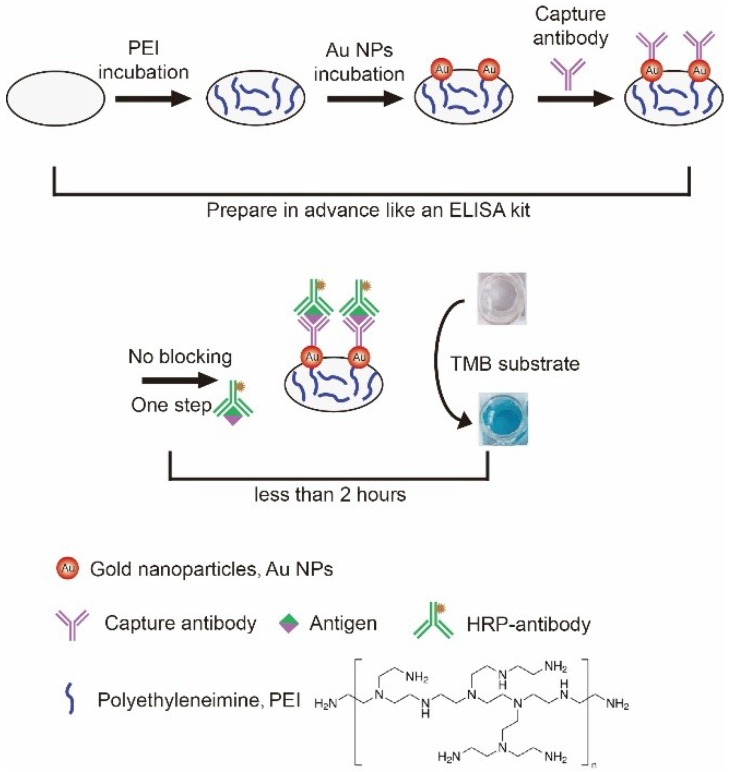
Schematic diagram of a PEI-GNP coated microwell plate and its application in a blocking-free “sandwich” ELISA.

In this paper, carcinoembryonic antigen (CEA) is the model target biomarker in our system, which has relationships with several kinds of cancer [24]. We combine the properties of gold nanoparticles (GNPs) and polyethyleneimine (PEI) polymers to construct a PEI-GNP coated microwell plate. We maintain the advantages of ELISA such as its high precision and good linearity, and endow new characteristics, including free-blocking and a reduced operating time (Scheme 1).

## 2. Experimental Section 

### 2.1. Materials and Instruments

PEI (25,000 kDa) and tetrachloroauric acid (HAuCl_4_, 99.99%) were from Sigma-Aldrich (Merck KGaA, Darmstadt, Germany). Sodium citrate was received from Shanghai Macklin Biochemical Co., Ltd. (Shanghai, China) BSA, PBS buffer, and Tween 20 were bought from Sangon Biotech (Shanghai, China) Co., Ltd. TMB substrate was purchased from Beyotime (Shanghai, China) Co., Ltd. (Shanghai, China) Captured antibodies for CEA, CEA, and HRP-labelled antibodies were provided by Fapon Biotech Inc., as were commercial products used in the ELISA kit. All chemicals were used as received without further purification. The 96-well microplates were Corning^®^ 96 Well Clear Polystyrene High Bind Stripwell™ Microplates (NO. 2592). Transmission electron microscope (TEM) images were taken from JEM-1230 (NIPPON TEKNO, Tokyo, Japan). Zeta potentials (ζ) were recorded using a Zeta Sizer Nano ZS (Malvern Zetasizer 3000HS and He/Ne laser at 632.8 nm at a scattering angle of 90° at 25 °C). Optical densities at 520 nm and 620 nm (OD_520_ and OD_620_) were tested using a Synergy H1 microplate reader (BioTek, Winooski, VT, USA). The UV-Vis spectrum was detected by UV 1780 (SHIMADZU, Tokyo, Japan). The concentration of gold was measured by inductively coupled plasma mass spectrometry (ICP-MS, NexIon300X, PerkinElmer, Boston, MA, USA). Optical photographs were recorded by an ILCE-6000L camera (SONY, Tokyo, Japan).

### 2.2. Synthesis and Characterization of Citrate-Coated GNPs

We synthesized GNPs with two different sizes (about 13 nm and 37 nm) using citrate-mediated reduction of HAuCl_4_. The synthetic protocol for 13 nm GNPs is as below. An aqueous solution of 1.0 mM, 100 mL HAuCl_4_ was heated to the boil and then 38.8 mM, 10 mL of citrate sodium aqueous solution was added. The mixture was kept boiling for another 15 min and cooled to room temperature naturally. The synthetic method for 37 nm GNPs was a little different. An aqueous solution of 0.4 mM, 100 mL citrate sodium was heated to the boil and then 24.3 mM, 1 mL of HAuCl_4_ aqueous solution was added. The mixture was kept boiling for another 15 min and cooled to room temperature naturally. The two kinds of GNPs were stored in a 4 °C refrigerator for further application. We used TEM to characterize the morphology and size of GNPs and meanwhile recorded the UV-Vis spectrum.

### 2.3. Formation of a Citrate-Coated Gold Nanoparticle Layer (GNPL) on PS Microplate Wells

We used deionized water (DI water) to dissolve PEI at the concentration of 5 mg/mL. Then, 100 μL of PEI solution was added in every commercial PS microplate well directly, kept for 1 min at room temperature and discarded. The microplate was washed twice with 200 μL DI water for 30 s. Lastly we dropped 100 μL of GNP solution in each PEI coated well, kept it for 5 min, removed the GNP solution, and rinsed twice with 200 μL DI water for 30 s. The whole process for GNPL formation on the surface of the well was completed within 10 mins. We dissolved the GNPs on the microplate wells using 150 μL of chloroazotic acid and used ICP-MS to measure the amount of GNPs.

### 2.4. ELISA Protocol on Untreated and GNPL Coated 96 Well Microplates

The ELISA protocol was the same for both untreated and GNPL coated 96 well microplates. Captured antibody (20 μg/mL, 100 μL/well) was added in the microplates overnight at 4 °C or for 2 h at 37 °C. For the routine ELISA in need of blocking, the next step was the addition of BSA solution (5%, mass fraction) and incubation for 2 h at 37 °C. In our blocking-free ELISA we did not require this step. Next, 100 μL of the mixture with different concentrations of antigen (0, 0.52, 1.03, 2.06, 4.13, 8.25, 16.50, and 33.00 ng/mL) and 0.4 μg/mL of HRP-labelled antibody were both dropped in each well and incubated for 1 h at 37 °C. Different concentrations of antigen were received through gradient dilution. After the washing step by PBST solution (PBS buffer and 0.1% Tween 20) three times, 200 μL of the TMB substrate was added in each well and kept for 30 min at 37 °C. Then, absorbance of the solution was measured at 620 nm using the multi-well plate reader and pictures recorded using a camera.

## 3. Results and Discussion

### 3.1. Characterization of GNPs with Different Sizes and the GNP Layer on the Microplates

**Figure 1:**
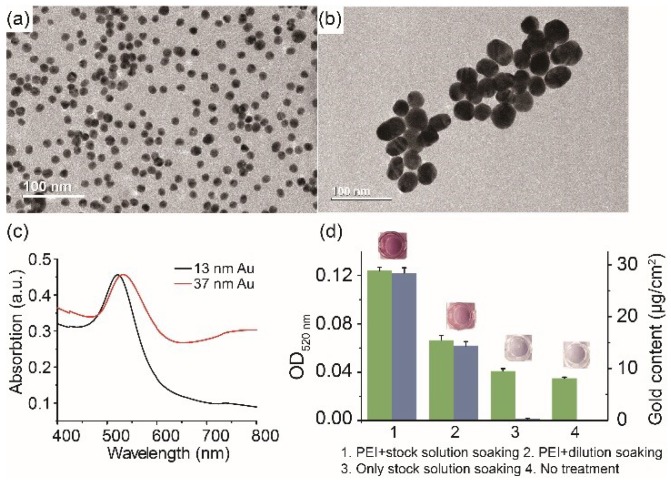
Characterization of gold nanoparticles (GNPs) and the GNP layer. Transmission electron microscope (TEM) image of 13 nm GNPs (**a**); TEM image of 37 nm GNPs (**b**); UV-Vis spectrum of GNPs (**c**); pictures with an optical density of 520 nm (OD_520_) of the gold content of microplates treated three different ways and untreated microplates (**d**).

We took advantage of sodium citrate to reduce tetrachloroauric acid, to synthesize GNPs. By changing the concentration and adding raw materials, GNPs with different sizes were received. We used TEM to characterize the morphology of GNPs, whose average diameters were 13 nm and 37 nm respectively (Figure 1a,b). These GNPs exhibited maximum absorbance at around 520 nm and 530 nm (Figure 1c). DLS results showed that GNPs were both negatively charged, with Zeta potentials of −37.4 eV and −12.9 eV. From TEM images and Zeta potentials, 13 nm GNPs had better dispersibility and a larger absolute value of Zeta potential, which meant better stability [25,26]. Therefore, at the beginning, we used 13 nm GNPs to form the layer.

We functionalized microplates with a GNP layer by using a PEI and GNP stock solution to steep them successively. GNPs attached to the surface of microplates via electrostatic interaction. To confirm whether GNPs adsorbed on the surface of microplates, after GNP incubation and washing, we recorded pictures with an optical density of 520 nm (OD_520_) to record the gold content of the microplate (Figure 1d). Obviously, after GNPs soak, the color of the microplate changes from transparent to red and the OD_520_ increased compared with that of untreated microplates. Meanwhile, we used two-fold dilution of GNPs to make the layer. The color was lighter and the OD_520_ was smaller when GNPs of a lower concentration were applied. We made use of chloroazotic acid to dissolve GNPs on the microplate for further quantifying the amount of gold using ICP-MS. The concentration of GNPs on the microplates was 29.12 μg·cm^−2^, 14.87 μg·cm^−2^, and 0 μg·cm^−2^ when the microplates were soaked with stock solution, two-fold dilution, and H_2_O separately. To verify the role of PEI in formation of the GNP layer, we added GNPs into microplates without the advanced PEI coating. Even after soaking for 24 h, only a small number of GNPs had adsorbed on the microplate surface, which was proven by pictures, OD_520_, and the result of ICP-MS (0.36 μg·cm^−2^). After successful functionalization of the GNP layer on the microplate, we investigated whether it has positive effects on the commercial ELISA.

### 3.2. Commercial ELISA on GNP Layer Coated Microplates

**Figure 2:**
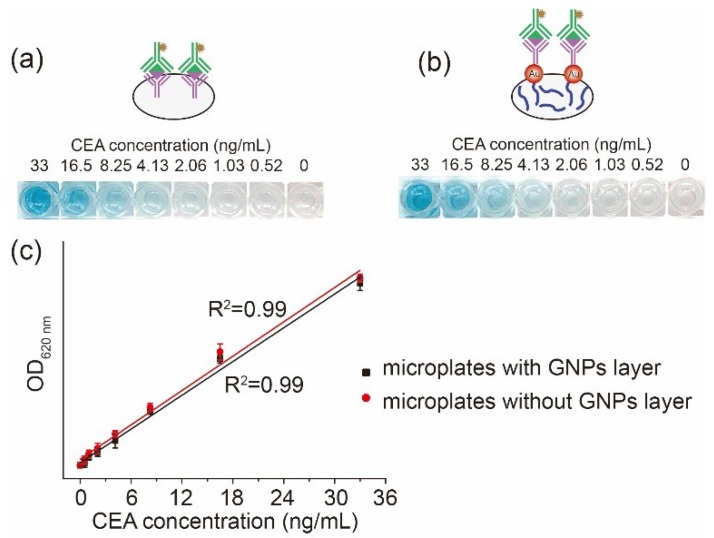
Results of the commercial ELISA. Pictures of the routine ELISA on the microplate without (**a**) and with (**b**) a 13 nm GNP layer. OD_620_ vs. CEA concentration of the routine ELISA on the microplate without and with a GNP layer (**c**).

To ensure the GNP layer on the microplate would not cause negative effects on the ELISA, we purchased commercial mature antibody and antigen (CEA) to perform the experiment. As a comparison, we did the same procedures on the untreated microplate. At the beginning, we only used microplates with a high concentration 13 nm GNP layer to test. After the steps of capturing the antibody coating, BSA blocking, antigen-HRP-labelled antibody complex incubation, and TMB substrates generating colored product, we recorded results through taking pictures (OD_620_). Almost no difference in result existed between the ELISA on the microplate with the GNP layer and without treatment (Figure 2). We analyzed sensitivity and linearity of CEA values from the assay on the microplate with the GNP layer, against those determined by the ELISA on the untreated microplate. For both conditions, OD_620_ increased with CEA concentrations becoming bigger (0, 0.52, 1.03, 2.06, 4.13, 8.25, 16.50, 33.00 ng·mL^−1^) and there was a linear range (0–33 ng·mL^−1^) with a strong correlation (R^2^ = 0.99) between OD_620_ and known CEA concentrations. These two assays revealed a 0.52 ng·mL^−1^ limit of detection (S/N = 3). After confirming that the GNP layer didn’t cause an adverse impact, we tried to explore whether it had positive effects on the ELISA. Considering there was no improvement in sensitivity of the ELISA, we decided to verify if introduction of the GNP layer can simplify procedures of the ELISA, for example, eliminating the blocking step that requires 2 h. Because PEI and citrate-GNPs were both hydrophilic and some literature has reported how the hydrophilic nature of some polymer-modified microplates can directly decrease nonspecific protein adsorption without any blocking step [18], we preformed the following exploration.

### 3.3. Blocking-Free ELISA on GNP Layer Coated Microplates

**Figure 3:**
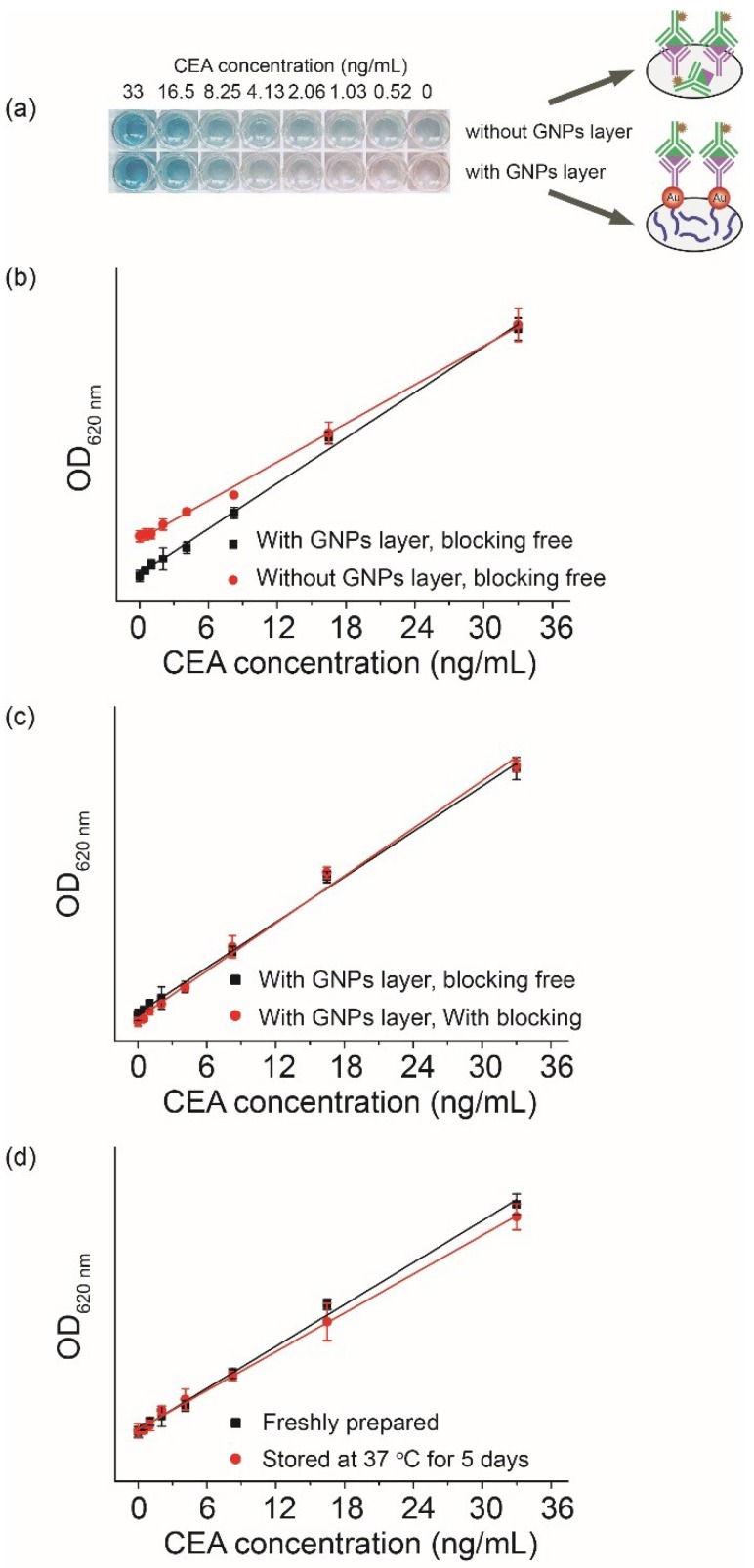
The results of the blocking-free ELISA. Pictures of the blocking-free ELISA on the microplate without and with the GNP layer (**a**); OD_620_ vs. CEA concentration of the blocking-free ELISA on the microplate without and with the GNP layer (**b**); OD_620_ vs. CEA concentration of the routine and blocking-free ELISA on the microplate with the GNP layer (**c**); OD_620_ vs. CEA concentration of the blocking-free ELISA on microplates with the GNP layer prepared freshly and stored at 37 °C over a period of five days (**d**).

Compared with the usual ELISA, a major advance of a blocking-free ELISA was elimination of the blocking step and an approximately 2 h saving. At the beginning, we applied microplates with a high concentration 13 nm GNP layer to test. To evaluate feasibility of the GNP layer in the blocking-free ELISA, on both microplates without and with the GNP layer, we performed the following steps: capture antibody coating, antigen-HRP-labelled antibody complex incubation (costing 1 h), and TMB substrates generating colored product (costing 30 min). If PEI–GNP coated microwell plates and immobilization of capture antibodies on the plate were prepared in advance such as in an ELISA kit, the whole process could take less than 2 h. We recorded results by taking pictures and collecting OD_620_ data. Obvious differences between the ELISA on the microplate with a GNP layer and without treatment existed (Figure 3a). At CEA concentrations of 33 and 16.5 ng·mL^−1^ the OD_620_ was almost the same. However, in the lower CEA concentration, OD_620_ in the microplate without the GNP layer was distinctly bigger than that in the microplate with the GNP layer (Figure 3b). Meanwhile, OD_620_ in the blocking-free ELISA on the microplate with the GNP layer, was nearly the same as the ELISA which had a blocking step on the microplate, also with the GNP layer (Figure 3c). When Figure 3b,c and Figure 2c are compared, the results display no unspecific adsorption of HRP-labelled antibody appeared on the microplate with the GNP layer and the GNP layer combined with blocking-free did not influence the ELISA, in which the limit of detection was still 0.52 ng·mL^−1^ (S/N = 3) and linearity was good (R^2^ = 0.99). Due to unspecific adsorption of HRP-labelled antibody on the untreated microplate in the blocking-free ELISA, OD_620_ in the CEA concentration of 0, 0.52, and 1.03 ng·mL^−1^ could not be distinguished. Using S/N = 3 as the standard, the limit of detection increased to about 2 ng/mL. Furthermore, to study stability of functionalized plates, accelerated stability testing was used. We prepared microwell plates with both a PEI–GNP layer and capture antibodies in advance, stored them at 37 °C over a period of five days, and carried out blocking-free ELISA using them. No obvious difference was presented between experiments using newly prepared microplates and those using stored microplates (Figure 3d). Referring to the literature [27], five days at 37 °C meant longer preservation time than six months at 2–8 °C, which showed acceptable stability.

### 3.4. The Influence of Different GNP Layer Coated Microplates on Blocking-Free ELISA

**Figure 4:**
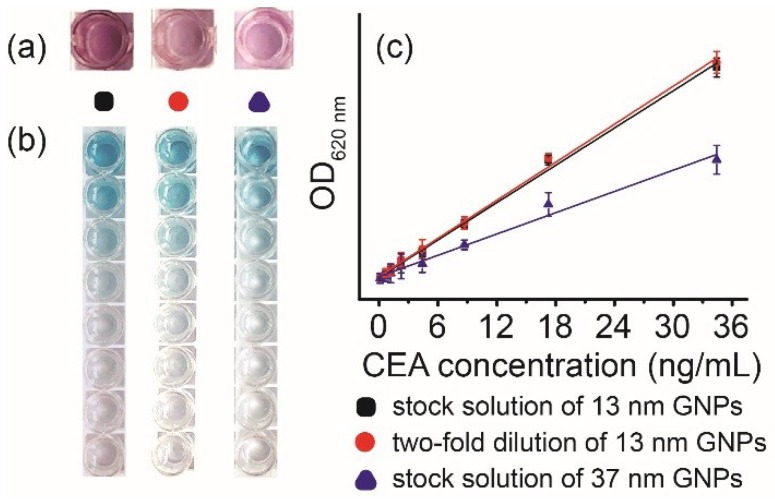
Results of blocking-free ELISA on different GNP layer coated microplates. Pictures of different GNP layers (**a**); pictures of blocking-free ELISA on three different GNP layers (**b**); OD_620_ vs. CEA concentration on three different GNP layers (**c**).

At the beginning, after PEI coating, we used stock solution of 13 nm GNPs to form the GNP layer and tried the above experiments. To investigate whether some parameters could influence effects of the GNP layer, we analyzed the impact of size and concentration of GNPs on the blocking-free ELISA. Two other GNP layers were prepared by two-fold dilution of 13 nm GNPs and stock solution of 37 nm GNPs, whose pictures are shown in Figure 4a. Blocking-free ELISA was performed on microplates with three kinds of GNP layer. After the procedure of capture antibody coating, antigen-HRP-labelled antibody complex incubation, and TMB substrates generating colored product, pictures and OD_620_ were recorded (Figure 4b,c). Neither concentration nor size of GNPs caused unspecific adsorption of HRP-labelled antibodies and blocking-free ELISA could be realized. However, the size of GNPs had an influence on OD_620_. On the layer of bigger GNPs (37 nm), OD_620_ was obviously lower than that on the layer of 13 nm GNPs, when CEA concentrations were between 8.25–33.00 ng·mL^−1^. However, at lower concentrations of CEA, this disparity was insignificant. The reason might be that it was easier for bigger GNPs to fall off the microplate, we therefore did the experiment to verify this. After the different sizes of GNP layers were washed with PBST ten times, chloroazotic acid was used to dissolve the GNPs left on microplates for further measurement by MS. The result for the 13 nm GNP layer decreased from 29.12 μg·cm^−2^ to 28.65 μg·cm^−2^, by contrast the result for the 37 nm GNP layer changed from 12.57 μg·cm^−2^ to 9.42 μg·cm^−2^. So more GNPs were lost when their size was bigger. Higher CEA concentrations represent more antigen-HRP-labelled antibody complex on the microplate and if GNPs fell off the surface, there was a higher HRP-labelled antibody loss. Therefore, on the 37 nm GNP layer, OD_620_ changed more obviously within a higher concentration range of CEA.

## 4. Conclusions

In summary, application of a GNP layer can save on the 2-h blocking step in a routine ELISA. The effects of 13 nm GNPs are better than that of bigger ones and using stock solution or two-fold dilution of GNPs (13 nm) to form the layer on the microplate doesn’t cause an obvious influence. If a PEI–GNP coated microwell plate and immobilization of capture antibodies on the plate are prepared in advance like an ELISA kit, with the addition of antigens, HRP-labelled antibodies (at the same time for 1 h), and TMB substrate colored reaction for 30 min, the whole ELISA process can be finished in less than 2 h. Less than 2 h from sample to answer is very time-effective, which can broaden application of the commercial ELISA and even have considerable prospect in the point of care test.
